# Causal Relationships Among Pollen Counts, Tweet Numbers, and Patient Numbers for Seasonal Allergic Rhinitis Surveillance: Retrospective Analysis

**DOI:** 10.2196/10450

**Published:** 2019-02-20

**Authors:** Shoko Wakamiya, Shoji Matsune, Kimihiro Okubo, Eiji Aramaki

**Affiliations:** 1 Institute for Research Initiatives Nara Institute of Science and Technology Ikoma Japan; 2 Graduate School of Science and Technology Nara Institute of Science and Technology Ikoma Japan; 3 Data Science Center Nara Institute of Science and Technology Ikoma Japan; 4 Musashikosugi Hospital Nippon Medical School Kawasaki Japan; 5 Nippon Medical School Hospital Nippon Medical School Bunkyo Japan

**Keywords:** seasonal allergic rhinitis, social media, Twitter, causal relationship, infoveillance, disease surveillance

## Abstract

**Background:**

Health-related social media data are increasingly used in disease-surveillance studies, which have demonstrated moderately high correlations between the number of social media posts and the number of patients. However, there is a need to understand the causal relationship between the behavior of social media users and the actual number of patients in order to increase the credibility of disease surveillance based on social media data.

**Objective:**

This study aimed to clarify the causal relationships among pollen count, the posting behavior of social media users, and the number of patients with seasonal allergic rhinitis in the real world.

**Methods:**

This analysis was conducted using datasets of pollen counts, tweet numbers, and numbers of patients with seasonal allergic rhinitis from Kanagawa Prefecture, Japan. We examined daily pollen counts for Japanese cedar (the major cause of seasonal allergic rhinitis in Japan) and hinoki cypress (which commonly complicates seasonal allergic rhinitis) from February 1 to May 31, 2017. The daily numbers of tweets that included the keyword “kafunshō” (or seasonal allergic rhinitis) were calculated between January 1 and May 31, 2017. Daily numbers of patients with seasonal allergic rhinitis from January 1 to May 31, 2017, were obtained from three healthcare institutes that participated in the study. The Granger causality test was used to examine the causal relationships among pollen count, tweet numbers, and the number of patients with seasonal allergic rhinitis from February to May 2017. To determine if time-variant factors affect these causal relationships, we analyzed the main seasonal allergic rhinitis phase (February to April) when Japanese cedar trees actively produce and release pollen.

**Results:**

Increases in pollen count were found to increase the number of tweets during the overall study period (*P*=.04), but not the main seasonal allergic rhinitis phase (*P*=.05). In contrast, increases in pollen count were found to increase patient numbers in both the study period (*P*=.04) and the main seasonal allergic rhinitis phase (*P*=.01). Increases in the number of tweets increased the patient numbers during the main seasonal allergic rhinitis phase (*P*=.02), but not the overall study period (*P*=.89). Patient numbers did not affect the number of tweets in both the overall study period (*P*=.24) and the main seasonal allergic rhinitis phase (*P*=.47).

**Conclusions:**

Understanding the causal relationships among pollen counts, tweet numbers, and numbers of patients with seasonal allergic rhinitis is an important step to increasing the credibility of surveillance systems that use social media data. Further in-depth studies are needed to identify the determinants of social media posts described in this exploratory analysis.

## Introduction

The rapid growth of the internet has been accompanied by an increase in the use of social media data (from sources such as Twitter and Facebook) to explore and understand various phenomena. This form of social media monitoring can facilitate an effective analysis of large quantities of social media data produced in real time.

Large-scale quantitative analyses have been conducted using health-related social media data [1,2], and the use of these data for disease surveillance (referred to as “infoveillance”) is gaining interest [3]. In particular, major advances have been made in the use of social media data to track the prevalence and spread of infectious diseases and other conditions [4-6]. These studies have contributed to public health by demonstrating moderately high correlations between fluctuations in the number of relevant social media posts and patients for a specific disease. Public health authorities have also started adopting and applying currently available tools that use social media for influenza surveillance, such as HealthTweets.org [7,8], Sickweather [9], and Now Trending [10].

Despite the reported correlations between actual disease prevalence and social media posts in previous research, the mechanism underlying this relationship is poorly understood. In other words, the causal relationship between disease occurrence and the behavior of social media users remains unclear. For example, some individuals may only begin posting on social media after an existing condition becomes more severe. In contrast, others may start posting when experiencing only mild symptoms and seek medical treatment after their conditions worsen. Due to the presence of these individual-level variations, the relationship between the numbers of social media posts and patient numbers remains inconclusive.

The lack of understanding of this relationship may have reduced the perceived reliability of disease surveillance based on social media data, as exemplified by the failure of Google Flu Trends [11]. This web service consistently overestimated influenza prevalence during the 2012-2013 season by over 50%, which led to a precipitous decline in its credibility as a surveillance system. The overestimations may have been influenced by the unusually early start of the 2012-2013 influenza season, which made it a frequent topic of discussion in many media outlets. As a consequence, this may have increased the number of people searching for influenza-related topics on Google. Although the system algorithm was eventually updated, Google Flu Trends was shut down in 2015. In order to increase the credibility of disease surveillance based on social media data, there is a need to determine if there is a causal relationship between the behavior of social media users and the actual number of patients for a target disease.

Seasonal allergic rhinitis is an allergic disease that is so widely prevalent in Japan that it can be considered a national affliction. In particular, a large number of patients suffer from seasonal allergic rhinitis induced by Japanese cedar pollen between February and April each year [12-14]. Although pollen is the main cause of seasonal allergic rhinitis, symptoms only occur if a person is exposed to a quantity of pollen that exceeds his/her threshold level. As a result, there is no strong association between pollen count and patient numbers. In addition, seasonal allergic rhinitis in Japan can also be triggered by pollen from other plant species (eg, hinoki cypress and common ragweed), which complicates disease surveillance. However, the predicted prevalence of seasonal allergic rhinitis in Japan is currently based solely on Japanese cedar pollen counts.

This study aimed to clarify the causal relationships among the prevalence of seasonal allergic rhinitis, the behavior of social media users, and the actions of the users in the real world. For example, we examined if more patients sought care after increases in pollen count, if Twitter users tweeted more after visiting a health care institute, and if more patients visited hospitals after seasonal allergic rhinitis received increased attention on social media. The results of this analysis may support the use of social media in seasonal allergic rhinitis surveillance and shed light on the previously unknown behavior of patients with seasonal allergic rhinitis. In addition, we discuss the content of some tweet examples.

## Methods

### Data

#### Measures

**Figure 1:**
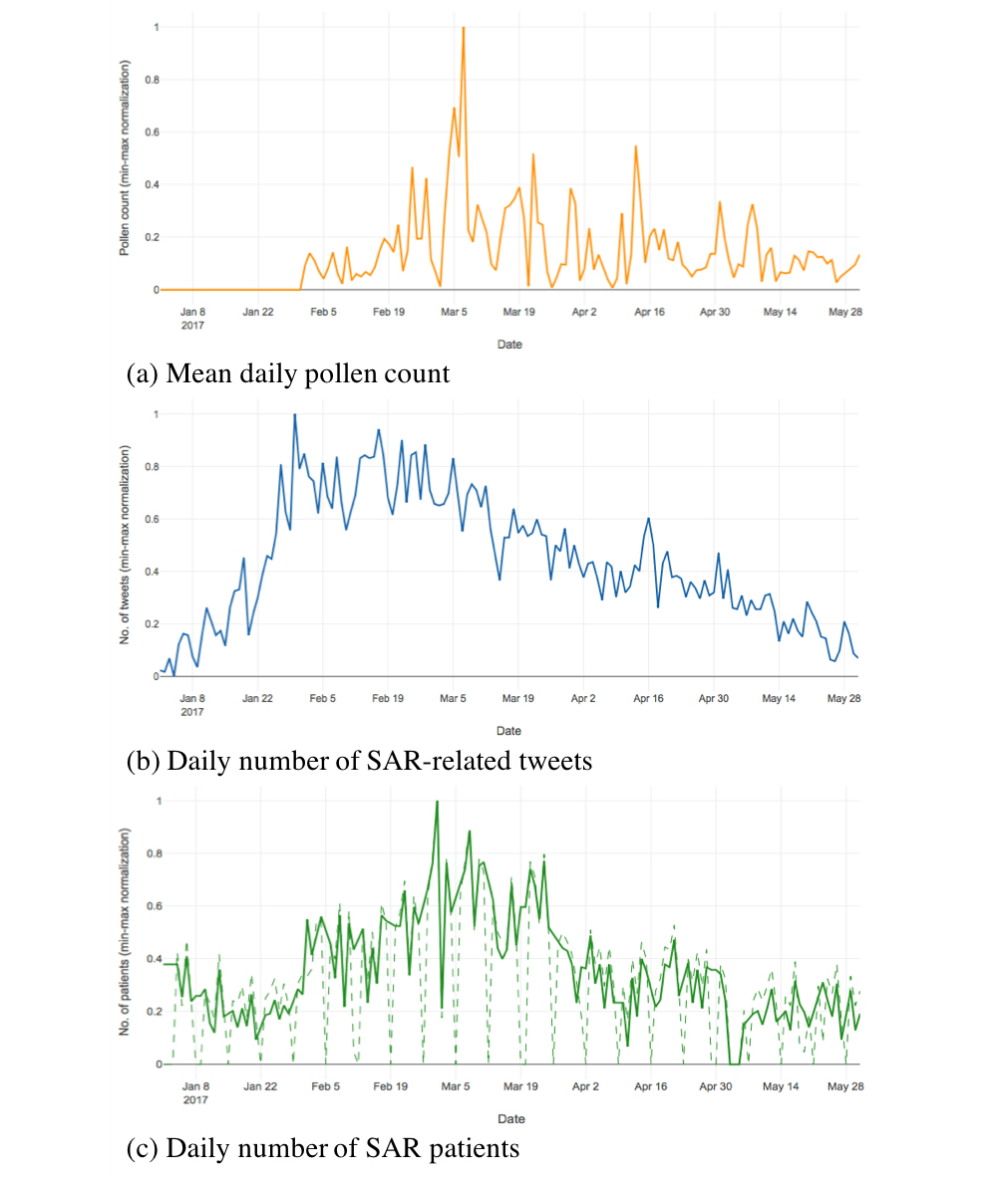
Time-based changes in data in Kanagawa Prefecture, Japan. The X-axes represent the date and the Y-axes represent data counts, to which min-max normalization is applied for the following variables: (a) changes in pollen count (mean daily pollen count from three observation sites within Kanagawa Prefecture), (b) changes in the number of SAR-related tweets, and (c) changes in the number of SAR patients (daily number of patients from three participating health care institutes within Kanagawa Prefecture). The solid line represents the changes in the number of patients on nonconsultation days (ie, days when an institute is closed) that were supplemented by the patient numbers from the preceding and proceeding days; the dashed line represents the changes in the reported number of patients. Our analysis used the supplemented patient numbers. SAR: seasonal allergic rhinitis.

This analysis was conducted using the following datasets of pollen count, tweets, and numbers of patients with seasonal allergic rhinitis (Figure 1). For this study, we focused on Kanagawa Prefecture in the Kantō region of Japan, which has the second largest population (over 9 million) in Japan, after Tokyo and before Osaka. The population density of Kanagawa Prefecture is estimated to be 3,791.56 people/km^2^ [15], which is ranked third in Japan, after Tokyo and Osaka. According to the report on the usage ratio of social networking services of internet users in each prefecture [16], Kanagawa Prefecture has a social networking services usage ratio of 56.5%, with the highest rank. According to the information by Kanagawa Prefectural Government [17], Japanese cedar pollen dispersal in Kanagawa Prefecture occurs from February to April each year, which corresponds with other areas in Kantō region including Tokyo and Saitama.

#### Pollen Count

We examined daily pollen counts for Japanese cedar (the major cause of seasonal allergic rhinitis in Japan) and hinoki cypress (which commonly complicates seasonal allergic rhinitis). Japanese cedar and hinoki cypress pollen counts are measured hourly by automatic pollen counters located nationwide. Each prefecture has two to three of these devices, which are placed in urban areas with high population densities and in mountainous regions that are the major source of pollen production. Each prefecture’s mean daily pollen count is calculated based on aggregated hourly counts from multiple observation sites.

Hourly pollen counts from February 1 to May 31, 2017, were obtained from the Japanese Ministry of the Environment’s pollen observation system (designated “Hanakosan”) for analysis [18,19]. The data were recorded at three observation sites within Kanagawa Prefecture (the Second Annex of the Kanagawa Prefectural Government Building, the Kawasaki Life Science & Environment Research Center, and the Kanagawa Environmental Research Center), and the mean daily pollen counts across these three sites were calculated. Figure 1 shows the changes in mean daily pollen count in Kanagawa Prefecture from February to May 2017.

#### Seasonal Allergic Rhinitis–Related Tweets Numbers

In an analysis of Twitter posts, we calculated the daily number of tweets that included the Japanese keyword “kafunshō” (or seasonal allergic rhinitis). These tweets have been crawled using the Twitter Streaming app’s programming interface. From among these tweets, we identified Twitter user profiles with location information (such as an area of residence that was freely written by a user in his/her Twitter profile or latitude/longitude data acquired from the satellite-based positioning systems such as global positioning system); the tweets were then classified according to the prefecture. We prioritized the latitude/longitude data, if available. Subsequently, we used retweets. We identified 185,538 tweets from Japan that contained the target keyword between January 1 and May 31, 2017. Figure 1 also shows the changes in the daily number of relevant tweets in Kanagawa Prefecture during this period.

#### Number of Patients With Seasonal Allergic Rhinitis

We analyzed the daily number of outpatients diagnosed with seasonal allergic rhinitis from January 1 to May 31, 2017. Specifically, electric health records were used to determine whether patients were diagnosed with seasonal allergic rhinitis, causing double counting of patients who saw a doctor twice or more often during the period, although this was a rare occurrence. Although daily patient numbers are not generally publicized, we obtained these data from three health care institutes that agreed to participate in the study. These institutes were Sasaki Hospital (Yokohama City), Kawasaki Saiwai Clinic (Kawasaki City), and Kosugi ENT Clinic (Kawasaki City). Figure 1 shows the changes in the daily number of seasonal allergic rhinitis patients that visited the participating health care institutes from January to May 2017. Single imputation methods such as last observation carried forward [20] were used to deal with missing values. However, missing values on nonconsultation days occurred regularly, and patients who did not want to wait until the following days may have visited a doctor on the preceding days. Thus, we interpolated the missing values from the average values.

### Analysis

We aimed to examine the causal relationships among the level of attention gained by seasonal allergic rhinitis on Twitter (number of tweets), pollen count, and the number of patients with seasonal allergic rhinitis. Although pollen counts may directly affect the number of tweets and patients with seasonal allergic rhinitis, the vice-versa is highly unlikely. As a result, we did not analyze the effects of tweet numbers and patient numbers on pollen count. Because this analysis used a data-driven approach, we employed the Granger causality test [21]. This statistical hypothesis test determines if a particular time series is predictive of another time series.

To determine if time-variant factors affect the causal relationships among seasonal allergic rhinitis prevalence, behavior of social media users, and the actions of users in the real world, we conducted additional analyses where the study period (February to May 2017) was divided into two phases. The first was the main seasonal allergic rhinitis phase, which generally occurs from February to April in the Kantō region for Japanese cedar-induced seasonal allergic rhinitis [12]. The second was the concluding phase of the season, which generally occurs in May in the Kantō region for Japanese cedar-induced seasonal allergic rhinitis. The data were analyzed as a differential time series of the differences between each day and the preceding day.

### Ethics Statement

This study utilizes only the participants’ count information that was nonlinkable, anonymized, and deidentified prior to analysis. As this research did not use personally identifiable information, it was exempt from institutional review board approval in accordance with the Ethical Guidelines for Medical and Health Research Involving Human Subjects stipulated by the Japanese national government.

## Results

### Overview

**Figure 2:**
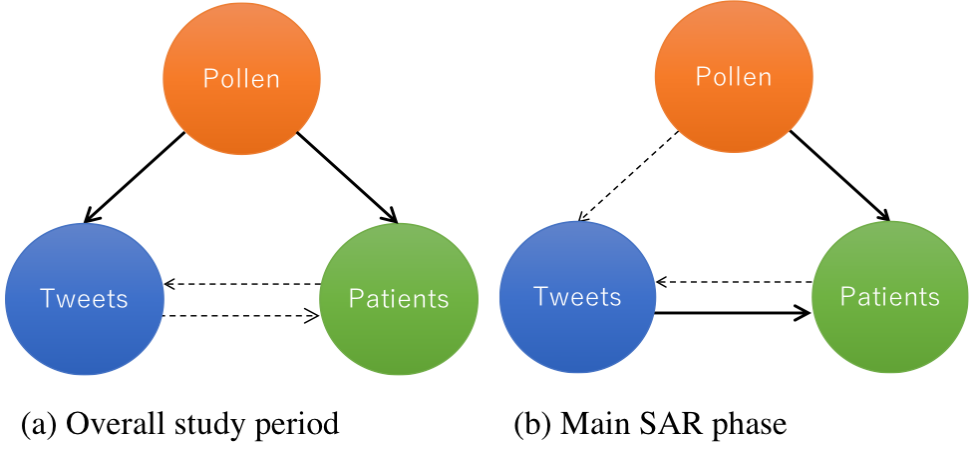
Causal relationships between pollen count, number of SAR-related tweets, and number of patients with SAR (Granger causality test results) for (a) the overall study period and (b) the main SAR phase. The Granger causality test did not reveal any causal relationships between these variables in the concluding phase of the season. SAR: seasonal allergic rhinitis.

In addition to analyzing the overall study period (February to May 2017), the Granger causality test was applied to the main seasonal allergic rhinitis phase (February to April 2017). The results of the overall study period and the main seasonal allergic rhinitis phase are presented in Table 1 and Figure 2.

### Effect of Pollen Count on the Number of Seasonal Allergic Rhinitis–Related Tweets

As shown in Table 1 and Figure 2, the Granger causality test rejected the null hypothesis that pollen count has no effect on the number of seasonal allergic rhinitis–related tweets during the overall study period (*P*=.04); this indicates that pollen count has a causal effect on the number of seasonal allergic rhinitis–related tweets. In contrast, the test did not reject this null hypothesis in the main seasonal allergic rhinitis phase (*P*=.05; Table 1; Figure 2). We were unable to apply the Granger causality test to the concluding phase (May 2017) for these two variables.

### Effect of Pollen Count on Patient Numbers

As shown in Table 1 and Figure 2, the Granger causality test rejected the null hypothesis that pollen count has no effect on patient numbers during the overall study period (*P*=.04); this indicates that pollen count has a causal effect on the number of seasonal allergic rhinitis–related tweets. In addition, the test also rejected the null hypothesis in the main seasonal allergic rhinitis phase (*P*=.01; Table 1; Figure 2). We were unable to apply the Granger causality test to the concluding phase (May 2017) for these two variables. Consequently, we confirmed that more patients sought care after increases in pollen count occurred during the overall study period.

**Table 1:** Results of the Granger causality test for pollen count, number of seasonal allergic rhinitis–related tweets, and number of patients with seasonal allergic rhinitis.

Cause/effect		*P* value	
		Number of tweets	Number of patients
**Overall study period (February to May 2017)**			
	Pollen count	.04	.04
	Number of tweets	—^a^	.89
	Number of patients	.24	—
**Main seasonal allergic rhinitis phase (February to April 2017)**			
	Pollen count	.05	.01
	Number of tweets	—	.02
	Number of patients	.47	—

^a^Not available.

### Effect of the Number of Seasonal Allergic Rhinitis–Related Tweets on Patient Numbers

As shown in Table 1 and Figure 2, the Granger causality test did not reject the null hypothesis that tweet numbers have no effect on patient numbers during the overall study period (*P*=.89). In contrast, the test rejected this null hypothesis in the main seasonal allergic rhinitis phase (*P*=.02; Table 1; Figure 2); this indicates that the number of seasonal allergic rhinitis–related tweets have a causal effect on patient numbers during this phase. We were unable to apply the Granger causality test to the concluding phase (May 2017) for these two variables. Therefore, we confirm that more patients visited hospitals after seasonal allergic rhinitis received increased attention on social media during the main seasonal allergic rhinitis phase.

### Effect of Patient Numbers on the Number of Seasonal Allergic Rhinitis–Related Tweets

In both the overall study period and the main seasonal allergic rhinitis phase, the Granger causality test did not reject the null hypothesis that patient numbers have no effect on the number of seasonal allergic rhinitis–related tweets (*P*=.24 and *P*=.47, respectively). We were unable to apply the Granger causality test to the concluding phase (May 2017) for these two variables.

## Discussion

### Content of Seasonal Allergic Rhinitis–Related Tweets

In this analysis of the 2017 Japanese cedar pollen-induced seasonal allergic rhinitis season in Kanagawa Prefecture, our results indicated that the level of attention gained by seasonal allergic rhinitis on Twitter and pollen count may be able to predict the number of patients with seasonal allergic rhinitis. In addition, we examined the content of seasonal allergic rhinitis–related tweets posted during the study period (January to May 2017) to gain further insight into these relationships.

**Figure 3:**
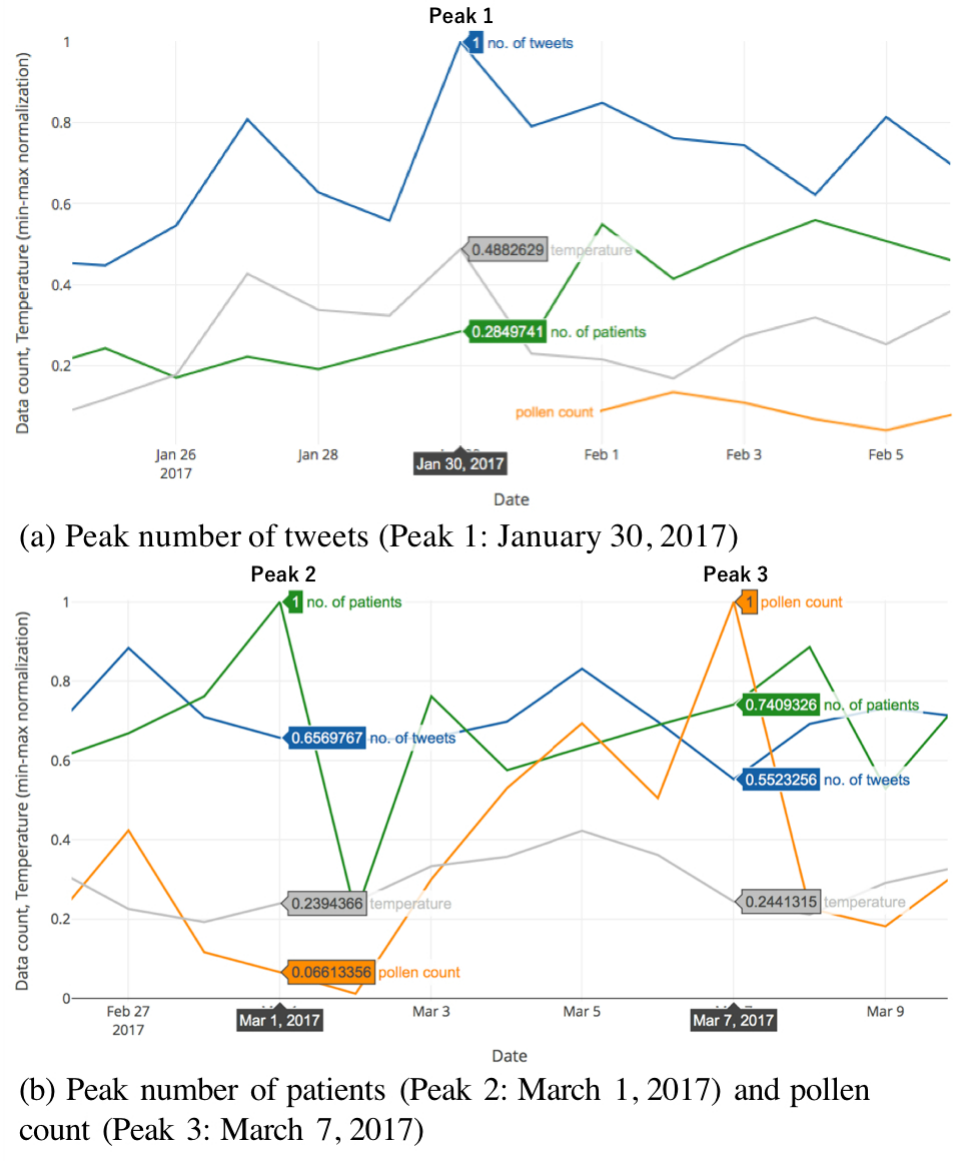
Peak number of SAR-related tweets, number of SAR patients, and pollen count. The X-axes represent the date and the Y-axes represent the min-max normalized data values for the three variables. The blue line represents the number of tweets, the green line represents the number of patients, the orange line represents the pollen count, and the grey line represents the mean temperature. SAR: seasonal allergic rhinitis.

The tweets in Textbox 1 are examples that were posted on January 30, 2017, which had the highest number of seasonal allergic rhinitis–related tweets before the pollen count, and the number of patients with seasonal allergic rhinitis increased. Pollen count is thought to increase in response to an increase in temperature and a decrease in humidity. In accordance with the expectations, there was a sudden increase in the ambient temperature on January 30, 2017 (Figure 3), which may have caused more sensitive users to identify and report symptoms ascribed to seasonal allergic rhinitis. This, in turn, may have led to a high level of attention to this topic on Twitter on that day.

The tweets in Textbox 2 are examples that were posted on March 1, 2017, which had the highest number of patients with seasonal allergic rhinitis seeking care at the three participating health care institutes during the study period. Coincidentally, there was an extremely low pollen count on this day. Although there appeared to be a slight decrease in tweet numbers (Figure 3), the tweets included those from patients with seasonal allergic rhinitis who were going or had gone to seek treatment.

Finally, the tweets in Textbox 3 are examples that were posted on March 7, 2017, which had the highest pollen count during the 2017 seasonal allergic rhinitis season. As shown in Figure 3, there was a sudden increase in pollen count for several days before the peak on this day. Although there was a reduction in the number of tweets, the tweets included those from people who had seasonal allergic rhinitis symptoms for the first time this season as well as reports of the worst symptoms for this season. There was also an increase in the number of patients with seasonal allergic rhinitis, and we confirmed that there were tweets where patients reported seeking treatment at health care institutes.

### Different Characteristics of Variables

Through our analysis of pollen count and tweet numbers, we were able to observe differences in data characteristics between these variables. The pollen count was affected by external factors (such as temperature, rainfall, wind speed, and wind direction), and there were substantial fluctuations throughout the study period (Figure 1). Accordingly, it was difficult to predict at a glance when the pollen season would end. On the other hand, the number of tweets showed some fluctuations (Figure 1), but there was a general increase from January, a peak in February and March, and a steady downward trend thereafter. Thus, we were able to visualize the trend toward the conclusion of the seasonal allergic rhinitis season. Our analysis showed that the combined use of data with different characteristics not only provided information on the prevalence of seasonal allergic rhinitis but also enabled observation of the seasonal allergic rhinitis season as it progressed.

The development of an analytical model that accounts for the different characteristics of the datasets while providing insight into the causal relationships may enable highly reliable disease surveillance.

**Examples of tweets posted on January 30, 2017.**

- I’ve been down with hay fever since that windy day. I don’t need this lol.
- Pollen is here, isn’t it? My hay fever’s not that bad, but I could feel it’s “arrival” 2/3 days ago. This morning I was hard-core sneezing and my nose can’t stop running...
- Looks like it’s hay fever.
- Good morning. I keep hearing about hay fever these few days…everyone’s most hated season is coming again, eh? You gotta eat lotus roots! Have a great day, everyone!
- Uh oh. I haven’t got any tissues. I’m dying. My runny nose won’t stop. I’m about to have hay fever. This is gonna be rough.
- I’m totally convinced that once it starts to get warm, then hay fever comes along.
- I haven’t got hay fever, but I can kind of feel the pollen flying.
- When it suddenly gets warmer I can feel the hay fever coming
- I don’t know if its hay fever or just the temperature differences in winter…but I’m snuffling.
- Today, it’s warm and windy, and I’m sneezing lots — is the pollen flying? I also heard that this year’s pollen count is 4.4 times more than last year’s. I already had a pretty rough time last year with my severe hay fever…stuffy nose, itchy eyes...I hate this. During this period I’d like to rip out my nose and eyeballs. Seriously.
- I hate this my nose won’t stop running, I don’t even know if this is the remnants of a cold or the effects of hay fever for real lol.
- I kind of feel like this year’s hay fever is already starting. I’ll stock up on OTC meds tomorrow.
- It...it’s finally here...hay fever, my eyes are itching and my nose is running and my head is heavy...it’s usually empty tho.
- My face is suddenly swollen today, and my eyes are bleary. it’s hay fever!!!
- I’m home! maybe it’s the warm weather, but I feel a bit hay fever-ish...what a pain
- When it’s this warm, hay fever’s here---- #nhk11

**Examples of tweets posted on March 1, 2017.**

- I’m fine when I’m outside, but I suddenly get hay fever symptoms around midnight. What’s up with this delayed attack.
- I woke up cos I couldn’t breathe cos my nose was stuffed cos of hay fever.
- My sleeping time and concentration are dwindling away because of hay fever
- Argh. This hay fever headache is massive…
- OH NO. I forgot to take my hay fever meds.
- My eyes are totally red cos of hay fever, But I still wanna use my colored contacts ~.
- I just took hay fever meds. I’ll sleep a bit more. I dunno why my shoulders are so stiff lately.
- This anti-hay fever mask has NO effect LOL
- I forgot my eye drops and nasal spray for hay fever but I haven’t got any symptoms. Maybe the non-drowsy oral meds are enough? Or is the real deal still to come?
- I’m using a mask but my nose is running from hay fever...
- Came to see the doctor for hay fever before work but it’s really crowded
- I’m here at the ENT. The doctor recommended an actual treatment to cure hay fever and not just suppress the symptoms but it looks like a pain to keep coming here. It seems I have to come here every month for 5 years, even in the off season. I want to do it if I have time next year!!
- So this is hay fever. If I go outside without a mask my nose becomes a waterfall...
- When I woke up today I was sniffing more than usual and my throat hurts, is this hay fever or a cold...if this continues tomorrow, I’ll probably come back tomorrow in even worse condition, last time I thought it was a cold and felt pretty confident, but it got worse and I lost my voice LOL

**Examples of tweets posted on March 7, 2017.**

- Hay fever sucks
- Good morning. Hay fever has arrived; my eyes and nose are so itchy~ I’ll try to be cheerful today as well.
- I’ve run out of hay fever meds, so if I seem to keep sneezing, I’ll probably have to get more meds from the doc.
- Oh man, my face is swollen and painful. Hay fever? Or maybe allergies?
- I’ve got hinoki hay fever and dust sensitivity, so I’ve GOT to have a mask for this old house in Yamanashi.
- My hay fever is going completely crazy today. Forgetting the tissues was a fatal mistake.
- Sudden spike in the number of people with hay fever symptoms!
- I thought I was sick, but the hospital told me I had hay fever — unbelievable...
- My hay fever is horrible today. This is the worst for this year.
- I just came back from the hospital, and I’ve gotta go again...my hay fever debut orz
- I was totally phased out with hay fever and almost forgot to go to school.
- My nose won’t stop running. I think it’s hay fever.
- My hay fever is crazy so I’ve got shades and a mask on. Totes feel like a celeb.
- Today’s hay fever is really rough. I’m completely done in with this triple play of sneezing, runny nose, and itchy eyes.
- The hay fever eye drops work only for just over 10 minutes. I really got to get some oral meds.
- I haven’t even stepped out of the house but the hay fever is so bad I don’t feel like doing anything.
- I’m sleepy already, this could also be the hay fever at work
- Hay fever where you don’t stop sneezing is a real pain.
- It’s not a cold, but my head hurts, and when that’s settling I’ve got a runny nose, am sneezing and coughing all at once. This is the start of legit hay fever.
- Been a while since I’ve had this full-blown hay fever. I blew my nose and it started bleeding, so I spent almost the whole day with a nosebleed. It won’t stop.
- The inside of my mouth itches cos of hay fever

### Limitations and Future Direction

This study is subject to several limitations, which could cause statistical and methodological limitations. First, we discuss the limitations caused by characteristics of social media data. This study assumed that there would be no lag effect in reporting symptoms on the same day on twitter. This study also assumed that the location where tweets originated would be the location that twitter users registered in their profile. Although ideally, only geolocation-enabled tweets must be used, the number of these tweets was small (less than 1% in our dataset) due to a recent increase in the number of users who care about their privacy and turn location sharing off. Notably, a lower quality of social media data is regarded as one of the common issues of most research using social media data. In addition, we used retweets as well as the other tweets because we assumed that retweeting tweets was one of the actions of Twitter users over social media. However, the retweet numbers tend to be significantly high when a public health agency or a celebrity tweets about an outbreak. Therefore, we should carefully consider the effect of a sudden increase in retweet numbers. Fortunately, existing research showed that the data-quality issue could be solved by the data quantity and applying appropriate preprocessing techniques to data such as location estimation [22,23], bot detection [24,25], positive/negative classification [26], and retweet handling [27]. In our future work, we plan to apply these techniques to our social media data to improve their quality.

In this analysis, we did not assess the effects of tweet numbers and number of patients with seasonal allergic rhinitis on pollen count because the number of tweets and patients would not directly affect the pollen count. Specifically, although tweet numbers and patient numbers are indicators of the behavior of social media users and their actual actions, pollen count observed outside is a natural phenomenon, which cannot be easily controlled by social media users. However, it will be possible to analyze the effects of tweet numbers and patient numbers on pollen count if the data observed indoor are available, because pollen count indoor can be controlled by people who take measures to remove pollen by, for example, using air cleaners. In our future work, we will try to analyze the unconsidered effects by gathering a variety of pollen count data and rule out any possible mathematical artefacts in an empirical fashion.

Further in-depth studies are needed to identify the determinants of social media posts described in this exploratory analysis. In addition, this study focused on one seasonal allergic rhinitis season, and downstream studies should be conducted using data that are continuously collected over multiple seasons. Furthermore, although the analyses in this paper were performed while focusing on one area due to the difficulty of obtaining the number of patients with seasonal allergic rhinitis, analyses using data that are collected in other areas should be conducted. Practically, it is very difficult to obtain the number of patients with seasonal allergic rhinitis in each area because no reports on seasonal allergic rhinitis patient numbers are provided in Japan and we have to identify health institutes (three or more in each area would be required) that can participate in this study. If we can obtain the patient numbers in other areas, we will conduct further analyses to interpret the results such as population density-based subgroup analysis. Finally, there is a need to conduct intervention-based prospective studies to gain a more accurate understanding of the causal relationships among these variables.

### Comparison With Prior Work

Health-related social media data have been used for large-scale quantitative analyses [1,2], referred to as “infoveillance” [3]. In particular, major advances have been made in the use of social media data to track the prevalence and spread of infectious diseases and other conditions. Among the infectious diseases targeted for surveillance, researchers have most actively applied these data to influenza surveillance [26-34]. Surveillance studies have also been conducted on enterohemorrhagic *Escherichia coli* outbreaks [35] and dengue fever [36]. In addition, social media data have been used to improve our understanding of Ebola [37] and Zika virus infections [38-40]. Although disease surveillance efforts tend to focus on acute infectious diseases, studies have also been conducted on chronic diseases such as cancer [41], hypertension [41], asthma [41-43], diabetes [44], and seasonal allergic rhinitis [45-47]. Systematic reviews have also been conducted on disease surveillance based on social media data [4-6].

Although these previous researches reported correlations between actual disease prevalence and social media posts, the mechanism underlying this relationship is still not investigated. In other words, the causal relationship between disease occurrence and the behavior of social media users remains unclear. Thus, this paper clarified the causal relationships among multisource data such as pollen count, the posting behavior of social media users, and the number of patients with seasonal allergic rhinitis in the real world, which is a novel point of this study compared with previous work.

### Conclusions

Although social media data are increasingly used in disease surveillance, there is a need to improve the credibility of these surveillance systems in order to promote their implementation and acceptance in society. Understanding the causal relationships between the behavior of social media users and actual patient numbers is an important step to increase the credibility of these surveillance systems. In this study, we analyzed data on pollen count, the number of tweets, and the number of patients during the 2017 seasonal allergic rhinitis season in Japan using the Granger causality test and shed light on the causal relationships among these variables. Increases in pollen count were found to increase the number of tweets and patients. In addition, increases in the number of social media posts (ie, tweets) also increased the patient numbers, suggesting that patients with seasonal allergic rhinitis or nonseasonal allergic rhinitis were motivated by increases in social media posts and went to the hospital. The main seasonal allergic rhinitis phase and the concluding phase of the season appeared to have different characteristics. Accordingly, disease surveillance based on social media data should be adjusted to account for these time-based differences.
